# TWO NATIVE SOUTH AMERICAN MALE POPULATIONS EXHIBIT CROSS-SECTIONAL INCREASE IN REGIONAL BRAIN VOLUME WITH AGE

**DOI:** 10.1093/geroni/igad104.3676

**Published:** 2023-12-21

**Authors:** Phoebe Imms, Nikhil Chaudhari, Nahian Chowdhury, Margaret Gatz, Michael D Gurven, Caleb Finch, Andrei Irimia, The Tsimane Health and Life History Project

**Affiliations:** University of Southern California, Los Angeles, California, United States; University of Southern California, Los Angeles, California, United States; University of Southern California, Los Angeles, California, United States; University of Southern California, Los Angeles, California, United States; University of California, Santa Barbara, Santa Barbara, California, United States; University of Southern California, Los Angeles, California, United States; University of Southern California, Los Angeles, California, United States

## Abstract

Features of industrialized environments such as consumption of processed foods and sedentarism are associated with faster brain atrophy. Studying brain volume trends with age in non-industrialized populations can elucidate environmental correlates of brain health. Indigenous to the Bolivian Amazon, the Tsimane and Moseten (T&M) are highly active forager-horticulturalists with some of the lowest rates of dementia. Tsimane have minimal interaction with mainstream Bolivians; Moseten are semi-acculturated. Age-related trends in regional brain volumes were estimated from T&M’s (N = 1,024; age range: 46-83) computed tomography and compared to trends estimated from Britons’ magnetic resonance images (UK Biobank, N = 19,973; same age range). In T&M males, parietal and occipital structures mediating visuospatial abilities exhibit small but significant rates of volume increase with age. Structures recruited during visuospatial navigation are important for survival in Amazonian rainforests. Frontal and temporal structures whose volumes decrease with age in Britons do not trend negatively with age in T&M males. T&M females, however, exhibit steeper cross-sectional rates of volume decrease with age compared to their industrialized counterparts. This suggests that brain volume trajectories, which can reflect dementia risk, are impacted by sex-specific aspects of non-industrialization. Despite T&M females’ higher rates of regional brain volume decrease with age compared to UK females, dementia rates in T&M females remain extremely low. Trends demonstrating increases in brain volume with age, such as those observed in T&M males, have not been reported in industrialized populations, suggesting that factors associated with being a male forager-horticulturalist may constrain regional brain atrophy.

